# Prognostic value of cardiovascular magnetic resonance left ventricular volumetry and geometry in patients receiving an implantable cardioverter defibrillator

**DOI:** 10.1186/s12968-021-00768-7

**Published:** 2021-06-10

**Authors:** Camila M. Urzua Fresno, Luciano Folador, Tamar Shalmon, Faisal Mhd. Dib Hamad, Sheldon M. Singh, Gauri R. Karur, Nigel S. Tan, Iqwal Mangat, Anish Kirpalani, Binita Riya Chacko, Laura Jimenez-Juan, Andrew T. Yan, Djeven P. Deva

**Affiliations:** 1 Department of Medical Imaging, St. Michael’s Hospital, Unity Health Toronto, University of Toronto, Toronto, Canada; 2 Radiology Department, Hospital de Clínicas de Porto Alegre, Porto Alegre, RS Brazil; 3 Schulich Heart Program, Sunnybrook Health Sciences Centre, University of Toronto, Toronto, Canada; 4 Joint Department of Medical Imaging, University Health Network, University of Toronto, Toronto, Canada; 5 Division of Cardiology, St. Michael’s Hospital, Unity Health Toronto, University of Toronto, Toronto, Canada; 6 Department of Medical Imaging, Sunnybrook Health Sciences Centre, University of Toronto, Toronto, Canada; 7 Li Ka Shing Knowledge Institute, Unity Health Toronto, Toronto, Canada; 8 St. Michael’s Hospital, 30 Bond Street, Toronto, M5B 1W8 Canada

**Keywords:** Cardiovascular magnetic resonance, Implantable cardioverter defibrillator, Sudden cardiac death, Left ventricular ejection fraction, Papillary muscles, Left ventricular sphericity

## Abstract

**Background:**

Current indications for implantable cardioverter defibrillator (ICD) implantation for sudden cardiac death prevention rely primarily on left ventricular (LV) ejection fraction (LVEF). Currently, two different contouring methods by cardiovascular magnetic resonance (CMR) are used for LVEF calculation. We evaluated the comparative prognostic value of these two methods in the ICD population, and if measures of LV geometry added predictive value.

**Methods:**

In this retrospective, 2-center observational cohort study, patients underwent CMR prior to ICD implantation for primary or secondary prevention from January 2005 to December 2018. Two readers, blinded to all clinical and outcome data assessed CMR studies by: (a) including the LV trabeculae and papillary muscles (TPM) (trabeculated endocardial contours), and (b) excluding LV TPM (rounded endocardial contours) from the total LV mass for calculation of LVEF, LV volumes and mass. LV sphericity and sphere-volume indices were also calculated. The primary outcome was a composite of appropriate ICD shocks or death.

**Results:**

Of the 372 consecutive eligible patients, 129 patients (34.7%) had appropriate ICD shock, and 65 (17.5%) died over a median duration follow-up of 61 months (IQR 38–103). LVEF was higher when including TPM versus excluding TPM (36% vs. 31%, *p* < 0.001). The rate of appropriate ICD shock or all-cause death was higher among patients with lower LVEF both including and excluding TPM (*p* for trend = 0.019 and 0.004, respectively). In multivariable models adjusting for age, primary prevention, ischemic heart disease and late gadolinium enhancement, both LVEF (HR per 10% including TPM 0.814 [95%CI 0.688–0.962] *p* = 0.016, vs. HR per 10% excluding TPM 0.780 [95%CI 0.639–0.951] *p* = 0.014) and LV mass index (HR per 10 g/m^2^ including TPM 1.099 [95%CI 1.027–1.175] p = 0.006; HR per 10 g/m^2^ excluding TPM 1.126 [95%CI 1.032–1.228] p = 0.008) had independent prognostic value. Higher LV end-systolic volumes and LV sphericity were significantly associated with increased mortality but showed no added prognostic value.

**Conclusion:**

Both CMR post-processing methods showed similar prognostic value and can be used for LVEF assessment. LVEF and indexed LV mass are independent predictors for appropriate ICD shocks and all-cause mortality in the ICD population.

## Introduction

The implantable cardioverter-defibrillator (ICD) has been established as an effective therapy for both primary and secondary prevention of sudden cardiac death (SCD) [1, 2]. Previous episodes of ventricular tachycardia or ventricular fibrillation, symptomatic heart failure with reduced left ventricular (LV) ejection fraction (LVEF) are class I indications for ICD implantation [1, 2]. However, contemporary data indicate that LVEF is a poor predictor of ICD treatment benefit [3–5]. Furthermore, ICD placement is costly, and inappropriate ICD shocks are associated with increased all-cause mortality [6]. Hence, there is a need for better risk stratification for SCD, to guide ICD treatment decision [7].

Balanced steady state free precession (bSSFP) cine imaging is considered the gold standard for morphological and functional LV assessment. When measuring LV function and mass using cardiovascular magnetic resonance (CMR), the trabeculae and papillary muscles (TPM) are usually considered part of the blood pool for better reproducibility. However, these correspond to myocardial tissue and theoretically should be included in the total LV mass (LVM) calculation [8]. Due to the technical challenges of delineating the TPM and lack of a universal automated contour algorithm, both methods are currently accepted for daily clinical practice [8]. Studies have analyzed differences between these two methods, concluding that measurements including TPM in the total LVM result in significantly smaller LV volumes and higher LVM and LVEF [9–11]. Given that LVEF is a key determinant of ICD implantation, such differences in measurements may have profound prognostic and therapeutic implications. To our knowledge no studies have directly compared these two CMR measuring methods in ICD recipients, and determined which method would be a better predictor of events.

Adverse LV remodeling on 2D transthoracic echocardiography is also associated with ventricular arrhythmia [12, 13]. Specifically, the pattern of LV remodeling has been linked with life-threatening arrhythmias in patients with low LVEF [14, 15]. This raises the question of whether measures of LV size, sphericity and concentricity should also be considered when evaluating candidates for ICD implantation, beyond LVEF.

The main purpose of this study is to compare and evaluate the prognostic value of different CMR methods of measuring LVM, volume and ejection fraction, and to determine whether CMR-derived LV volumetric and geometric parameters provide additive prognostic value for appropriate ICD shock therapy or mortality.

## Methods

### Study population

Data from consecutive patients who underwent ICD implantation for primary or secondary SCD prevention at two university tertiary care hospitals (St. Michael’s Hospital, Toronto, Canada; Sunnybrook Health Sciences Center, Toronto, Canada) from January 2005 to December 2018 and had a CMR study before ICD implantation were retrospectively reviewed. Subject’s demographics and clinical follow-up were obtained from electronic patient records. From a total of 425 consecutive eligible patients, 20 with incomplete LV stack and poor image quality that precluded LV volumetric analysis were excluded, as well as 33 patients with different etiologies [Brugada syndrome (n = 6), long QT syndrome (n = 6), hypertrophic cardiomyopathy (n = 14), and arrhythmogenic right ventricular cardiomyopathy (n = 7)], for whom LVEF was not the primary determinant of ICD decision. The local institutional review board approved the study protocol, and due to its retrospective nature, waived the need for written informed consent.

All decisions regarding ICD device implant, pharmacologic therapy, and follow-up frequency were made at the discretion of the cardiologist responsible for the patient’s care. Patients were followed prospectively both in the device clinic at 6-month intervals and/or followed-up more urgently based on home-monitoring events. All events were reviewed by trained device technicians and by an attending electrophysiologist blinded to CMR measurements. Deaths were determined using electronic chart review, hospital and autopsy records, or confirmed by the primary care provider. Patients who did not develop the corresponding endpoint by the end of the observation period were censored at the last clinic follow-up. The primary endpoint was a composite of appropriate ICD shock or all-cause death. The secondary endpoint was all-cause mortality.

### Image acquisition and analysis

Images were obtained with a 1.5 T CMR scanner (Achieva, Philips Healthcare, Best, Netherlands; TwinSpeed Excite, General Electric Healthcare, Milwaukee, Wisconsin) or a 3 T scanner (Magnetom Skyra, Siemens Healthineers, Erlangen, Germany) using surface coils and retrospective electrocardiographic (ECG) triggering for capture of the entire cardiac cycle. All scans included LV cines (horizontal long-axis, vertical long-axis, 2-chamber, 3-chamber, 4-chamber, and short-axis) and late gadolinium enhancement (LGE). LV cine images were acquired using a bSSFP sequence (slice thickness 8 mm, gap 2 mm, temporal resolution < 40 ms, in-plane spatial resolution 2 × 2 mm). Gadolinium based contrast agents used included gadobenate dimeglumine (Multihance; Bracco Diagnostic, Inc, Milan, Italy), gadoteridol (Prohance; Bracco Diagnostic, Inc.), and gadobutrol (Gadovist; Bayer Healthcare, Berlin, Germany). LGE images were obtained 10–20 min after injection of gadolinium in a peripheral vein at a dose of 0.1–0.2 mmol/kg of body weight. Breath-hold short and long-axis 2D segmented inversion-recovery sequences (slice thickness 8 mm, gap 2 mm, TR 4.2 ms, TE 1.8 ms, FA 50**°**, FOV 320 × 320mm^2^, matrix 256 × 196, in-plane spatial resolution 2 × 2 mm) were used for LGE imaging.

**Fig. 1:**
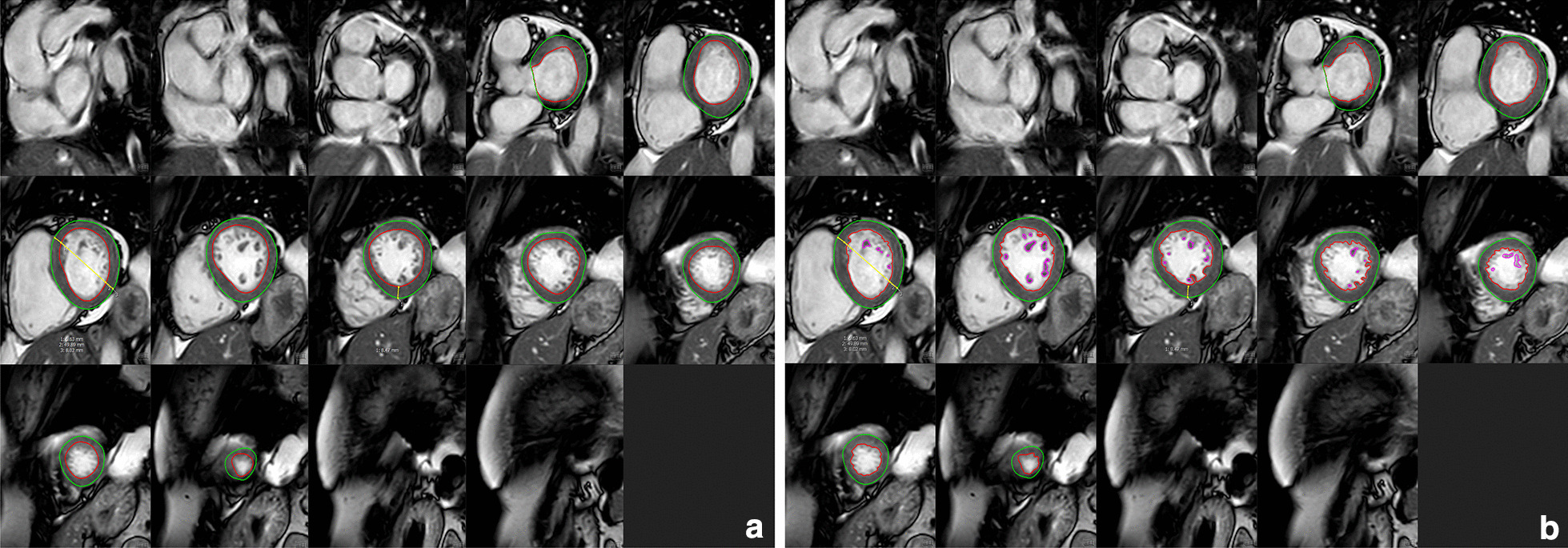
CMR post-processing methods for data acquisition. Endocardial left ventricular (LV) contours (in red) were drawn **a** excluding and **b** including trabeculae and papillary muscles (TPM) in the total LV mass as shown (in purple). Epicardial contours (in green) were maintained between techniques for LV mass calculation. LV end—diastolic diameter and anterior and posterior wall thickness were measured at mid ventricular level (yellow lines)

Two fellowship-trained radiologists independently evaluated and post-processed cine images using a commercial software (cvi^42^, version 5.11, Circle Cardiovascular, Calgary, Alberta, Canada), blinded to other clinical and outcome data. Two different methods were used to calculate LV end-diastolic volume (LVEDV), end-systolic volume (LVESV), stroke volume (SV), LVEF, and LVM from contiguous short-axis stack images (Fig. 1). Manual tracing of the endocardial borders at end-diastole and end-systole was performed, excluding TPM from the LVM, considering them part of the blood pool (rounded endocardial contours). The endocardial contour was erased and traced again at end-diastole and end-systole with blood pool thresholding to detect TPM, which were included in the total LVM (trabeculated endocardial contours). Epicardial borders were manually drawn on the external myocardial border at end-diastole, in the middle of the chemical shift artifact line if present. LVM including and excluding TPM was calculated as the sum of myocardial volume multiplied by the specific gravity (1.05 g/mL) of myocardial tissue. The anteroseptal and inferolateral myocardial wall thickness, as well as the end–diastolic cavity dimension, were measured on short-axis cine images at the tip of the papillary muscles.

To assess LV geometry, the LV long-axis diameter was measured in the 4-chamber cine, in systole and in diastole, from the center of an imaginary line at the base of the mitral valve to the LV apex. LV end-systolic and end-diastolic sphere volumes were calculated as the volume of a sphere using the end-systolic and end-diastolic long-axis diameters, respectively, using the following formula: 4/3*π*(D/2)^3^ [16]. The Sphericity Index was calculated as a ratio between the LVEDV and the volume of a sphere using the following formula: EDV/(4/3*π*(D/2)^3^), where D corresponds to the end-diastolic long axis diameter [16]. Sphericity indices were calculated for both segmentation methods, including and excluding trabeculae and papillary muscles. The assessment of LV remodeling was assessed based on the relative wall thickness (RWT) and LVM/volume ratio (M/V). RWT was calculated as the ratio of LV anterolateral plus inferolateral wall thicknesses to the end-diastolic cavity dimension. The M/V ratio was calculated by dividing LVM by LVEDV. All CMR indices (LVEDVI, LVESVI, LVMI, and LVES and LVED sphere volume indices) were indexed by body surface area (BSA) using the Mosteller formula.

### Statistical analysis

Analysis was performed using SPSS (version 22, Statistical Package for the Social Sciences, International Business Machines, Inc., Armonk, New York, USA) and STATA version 16.1 (Stata Corp, College Station, Texas, USA). Categorical variables were presented as frequencies and percentages, and continuous variables as mean and standard deviation or median and quartiles, as appropriate. Categorical variables were compared with Chi-square test or Fisher’s exact test. Non-parametric tests Mann–Whitney U test and Kruskal–Wallis test were used to compare continuous data between two and three groups, as appropriate. Relationships between CMR parameters were examined using non-parametric Spearman’s correlation test (Spearman’s rho). To determine inter-observer reproducibility, both readers independently measured a random sample of 20 CMRs. Intraclass correlation coefficients (ICC) for absolute agreement were calculated. Readers had a very good interobserver agreement, with ICC > 0.95 for all CMR measurements based on both methods. Intra-observer agreement was also excellent for all CMR parameters (ICC > 0.95) with exception of LVM, with both methods showing similar reproducibility (ICC = 0.94 for LVM excluding TPM and ICC = 0.98 for LVM including TPM).

A paired t-test was performed to compare the two CMR measurement methods (including TPM in, and excluding TPM from the total LVM), and 95% confidence intervals (95%CI) were calculated. Data on time to the primary endpoint across quartiles of the LV measurements were displayed by Kaplan–Meier curves and compared with the log-rank test for trend. Cox proportional hazards regression model was performed to assess the association between each CMR parameter and the primary endpoint. For multivariable analysis, LVMI, LVEDVI, and LVESVI were included in different models adjusting for clinically relevant variables, such as age, type of prevention (primary or secondary), ischemic etiology (versus non-ischemic) and presence of LGE. Stratification was used for variables not fulfilling the proportional hazard assumption. Model discrimination was measured by concordance statistic (Harrell's C) and Somers' D. Statistical significance was defined as a two-sided p-value < 0.05. To determine whether the predictive value of the 2 methods varies in various patient subgroups, we tested for interactions between LV parameters based on the two methods and clinical factors including primary versus secondary prevention, ischemic etiology, presence of LGE and QRS duration.

## Results

Of the initially identified 425 patients, a total of 372 patients were included in the final analysis. Of these, 294 patients were male (79%). The average age at ICD implantation was 60 years. Of the 372 patients, 221 (59.4%) had known ischemic heart disease and 238 (64%) received an ICD as primary prevention. The median time from pre-implantation CMR to ICD insertion was 31 days (IQR 5–155). Over a median follow-up of 61 months (IQR 38–103), 65 patients died (17.5%) and 129 patients experienced an appropriate ICD shock or death (34.7%). Detailed baseline demographic information of the study population is displayed in Table 1. The CMR measurements are summarized in Table 2.

**Table 1:** Baseline demographic characteristics of study population

Number of patients, N	372
Age at ICD implant, years, mean (SD)	61 (13)
Gender, n (%)	
Male	294 (79%)
Cardiovascular disease risk factors, n (%)	
Hypertension	194 (52%)
Dyslipidemia	180 (49%)
Diabetes	106 (29%)
Smoking History	
Unknown	17 (5%)
Never	194 (52%)
Previous	121 (33%)
Current	40 (11%)
Angina	51 (14%)
Myocardial infarction	156 (42%)
Heart failure	151 (41%)
Cardiac arrest	21 (6%)
Percutaneous coronary intervention	73 (20%)
Coronary artery bypass graft surgery	64 (17%)
Ischemic heart disease	221 (59%)
Stroke	28 (8%)
Chronic kidney disease	25 (7%)
Cardiovascular medications, n (%)	
Aspirin	203 (55%)
Adenosine diphosphate receptor inhibitors	54 (15%)
Anticoagulation	106 (29%)
Diuretic	165 (44%)
Beta blocker	298 (80%)
Calcium channel blocker	20 (5%)
Angiotensin-converting-enzyme inhibitors	237 (64%)
Angiotensin receptor blocker	45 (12%)
Statin	242 (65%)
Nitroglycerin	12 (3%)
Antiarrhythmic drugs	62 (17%)
Indication	
Primary prevention	238 (64%)
Secondary prevention	134 (36%)
Type of ICD device	
Single-chamber ICD	188 (50.7%)
Dual-chamber ICD	109 (29.4%)
Cardiac resynchronization therapy defibrillator	74 (19.9%)
ECG findings	
QRS duration, ms, mean (SD)	122 (31)
Intraventricular conduction abnormalities	
Left bundle-branch block	87 (29.4%)
Right bundle-branch block	26 (7.1%)
Non-specific intraventricular conduction delay	61 (16.6%)
Presence of late gadolinium enhancement (LGE)	266 (71.5%)
Ischemic LGE	179 (51%)

*ECG* electrocardiogram, *ICD* implantable cardioverter defibrillator, *LGE* late gadolinium enhancement

**Table 2:** Cardiovascular magnetic resonance parameters, mean (SD)

	Including TPM (trabeculated endocardial contours)	Excluding TPM (rounded endocardial contours)	
LVEDV, mL	237 (79)	275 (89)	
LVEDVI, mL/m^2^	122 (41)	142 (45)	
LVESV, mL	159 (76)	197 (88)	
LVESVI, mL/m^2^	82 (40)	102 (45)	
LVEF, %	36 (15)	31 (13)	
LVM, g	178 (52)	138 (41)	
LVMI, g/m^2^	91 (24)	71 (19)	
LVM/LVEDV ratio	0.79 (0.21)	0.52 (0.13)	
LV sphericity index	0.45 (0.11)	0.52 (0.13)	
LV end-diastolic sphere volume, mL			542 (161)
LV end-systolic sphere volume, mL			421 (169)
LV end-diastolic sphere volume index, mL/m^2^			280 (78)
LV end-systolic sphere volume index, mL/m^2^			218 (85)
Anteroseptal wall thickness, mm			9 (2)
Inferolateral wall thickness, mm			7 (2)
LV end-diastolic dimension, mm			66 (10)

*LV* left ventricle, *LVEDV* left ventricular end-diastolic volume, *LVEDVI* left ventricular end-diastolic volume index,  *LVESV* left ventricular end-systolic volume, *LVESVI* left ventricular end-systolic volume index, *LVEF* left ventricular ejection fraction, *LVM* left ventricular mass, *LVED* left ventricular end-diastolic, *LVES* left ventricular end-systolic, *I* index, *SD.* standard deviation, *TPM* trabeculae and papillary muscleas

### Relationship between LV measurements using two different post-processing methods

As compared with the method excluding TPM, LVEF was 5% (95% CI 4.7–5.3%, p < 0.001) higher, LVEDVI was 20 ml/m^2^ (95% CI 18.8–20.4 ml/m^2^) lower, and LVMI was 21 g/m^2^ (95% CI 20.0–21.5 g/m^2^) higher when TPM were included. LVEF was inversely correlated with LV sphericity index whether including (rho = − 0.47) or excluding TPM (rho = − 0.43); LVEDVi (rho = 0.54 and 0.50) and LVESVI (rho = 0.56 and 0.52) were moderately positively correlated with LV sphericity whether including or excluding TPM, respectively (all p < 0.001).

### Prognostic value of LV measurements

**Fig. 2:**
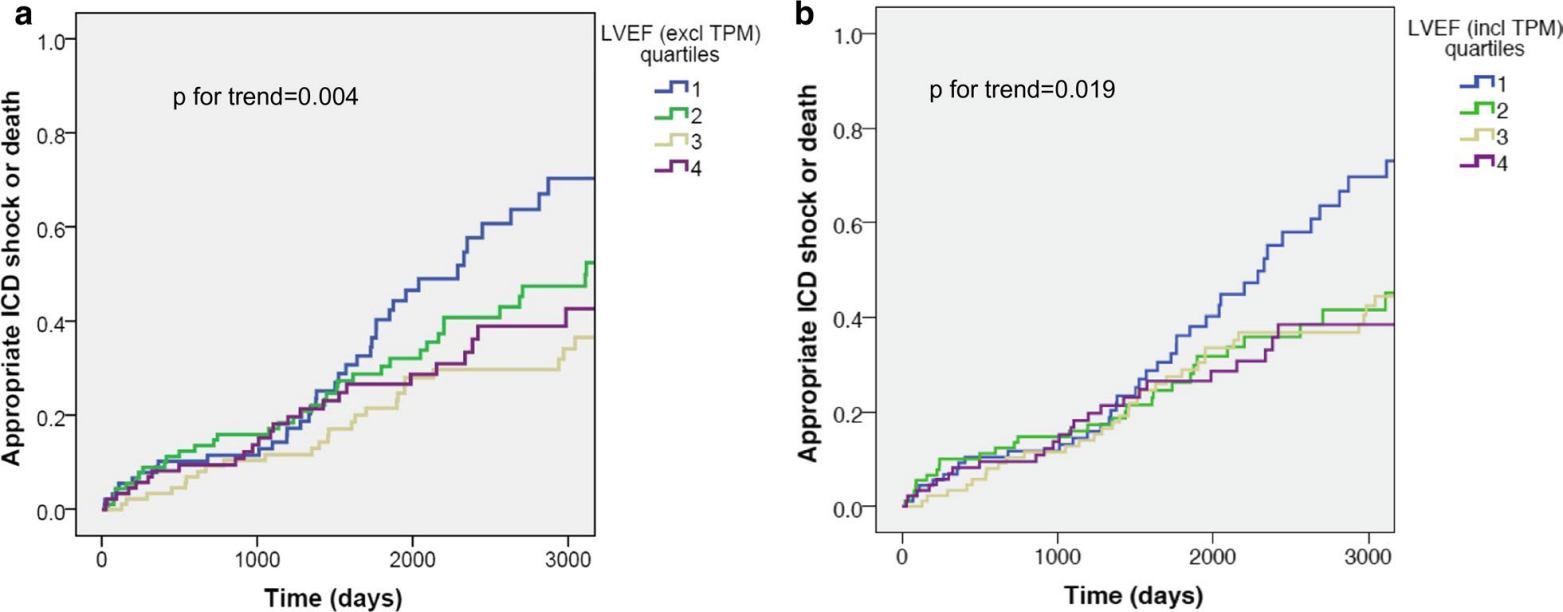
Kaplan–Meier curves for comparison of appropriate implanted cardioverter-defibrillator (ICD) shock or death between 2 CMR contouring methods. Left ventricular (LV) ejection fraction (LVEF) is displayed in quartiles, measured by **a** excluding and **b** including the trabeculae and papillary muscles (TPM) in the total LV mass. LVEF excluding TPM: median = 27.9 (25th percentile = 21.9, 75th percentile = 37.7); LVEF including TPM median = 32.9 (25th percentile = 25.2, 75th percentile = 44.3). Survival or event-free times were significantly longer for higher LVEF whether measured by excluding (log rank test, *p for trend* = 0.004) or including TPM (log rank test, *p for trend* = 0.019)

Over a median follow-up time of 61 months, the rates of appropriate ICD shocks or death were lower across ascending quartiles of LVEF including (*p* for trend = 0.019) and excluding TPM (*p* for trend = 0.004), as shown on Fig. 2. There was also a higher mortality rate for higher quartiles of LVEDVI including (*p* for trend = 0.011) and excluding TPM (*p* for trend = 0.017), higher quartiles of LV end-systolic sphere volume index (*p* for trend = 0.020), and higher quartiles of LV sphericity index including (*p* for trend = 0.003) and excluding TPM (*p* for trend = 0.001). No significant differences in the primary or secondary endpoints were observed across quartiles of LVM/LVEDV ratios.

LVESVI, LVMI and LVEF were significant predictors of appropriate ICD therapy or death, whether measured by including or excluding TPM from the total LVM. The hazard ratios for appropriate ICD shocks and death occurring were similar for both contouring methods, whether the LVEF cut point was 30% or 35%. The results of the univariable analysis are displayed in Table 3.

**Table 3:** Univariable analysis of evaluated CMR parameters for the prediction of appropriate implantable cardioverter defibrillator shock or death

	Hazard ratio	95% CI	P value
*Excluding TPM (rounded endocardial contours)*			
LVEDVI, per 10 ml/m^2^	1.029	0.997–1.063	0.079
LVESVI, per 10 ml/m^2^	1.039	1.005–1.074	0.025
LVMI, per 10 g/m^2^	1.104	1.025–1.189	0.009
LVEF, per 10%	0.805	0.683–0.951	0.011
LVEF ≤ 30%	1.526	1.055–2.206	0.025
LVEF ≤ 35%	1.240	0.823–1.869	0.304
LVM/LVEDV ratio	1.181	0.319–4.378	0.803
*Including TPM (trabeculated endocardial contours)*			
LVEDVI, per 10 ml/m^2^	1.030	0.993–1.068	0.11
LVESVI, per 10 ml/m^2^	1.040	1.002–1.081	0.040
LVMI, per 10 g/m^2^	1.081	1.021–1.145	0.008
LVEF, per 10%	0.836	0.726–0.962	0.012
LVEF ≤ 30%	1.533	1.087–2.162	0.015
LVEF ≤ 35%	1.383	0.964–1.984	0.078
LVM/LVEDV ratio	1.259	0.513–3.091	0.61
RWT	0.981	0.091–10.530	0.99

*CI* confidence interval, *LVEDVI* left ventricular end-diastolic volume index, *LVESVI* left ventricular end-systolic volume index, *LVEF* left ventricular ejection fraction, *LVMI* left ventricular mass index, *RWT* relative wall thickness, *TPM* trabeculae and papillary muscles

Multivariable analysis revealed that LVEF and LVMI had independent prognostic value after adjusting for age, ischemic heart disease and presence of LGE, with lower LVEF values (Harrell's C including TPM 0.588 and excluding TPM 0.590) and higher LVMi values (Harrell's C including TPM 0.597 and excluding TPM 0.600) predictive of increased ICD shocks and death. However, when assessing LVEF and LVMI in the same model, they did not show predictive value beyond each other, whether including (p = 0.30 and p = 0.053, respectively) or excluding TPM p = 0.17 and p = 0.062, respectively). Other variables such as LVESVI and LVEDVI, as well as LV sphericity were associated with increased mortality, but were not independent predictors of appropriate ICD therapy or death (p > 0.05). RWT was also not a significant predictor for the primary or secondary endpoint. The results for the multivariable models, including their concordance statistics, are depicted in Table 4. In the primary prevention subgroup, both methods for LVEF calculation showed similar prognostic value for our primary and secondary outcomes, similar to the entire study cohort.

**Table 4:** Multivariable analysis of CMR parameters for the prediction of appropriate implantable cardioverter defibrillator therapy or death

Variables	Adjusted HR	95% CI	p	Harrell's C	Somers' D
LVMI excluding TPM, per 10 g/m^2^	1.126	1.032–1.228	0.008	0.600	0.200
LVMI including TPM, per 10 g/m^2^	1.099	1.027–1.175	0.006	0.597	0.194
LVEDVI, excluding TPM, per 10 ml/m^2^	1.034	0.993–1.075	0.10	0.558	0.115
LVEDVI, including TPM, per 10 ml/m^2^	1.033	0.989–1.080	0.17	0.559	0.118
LVESVI excluding TPM, per 10 ml/m^2^	1.044	1.002–1.087	0.038	0.565	0.131
LVESVI, including TPM, per 10 ml/m^2^	1.044	0.998–1.093	0.061	0.569	0.139
LVEF excluding TPM, per 10%	0.780	0.639–0.951	0.014	0.590	0.181
LVEF including TPM, per 10%	0.814	0.688–0.962	0.016	0.588	0.175
RWT	0.901	0.069–11.710	0.936	0.561	0.122

All models adjusted for age, primary prevention, ischemic heart disease, and presence of late gadolinium enhancement

*HR* hazard ratio, *CI* confidence interval, *LVMI* left ventricular mass index, *LVEDVI* left ventricular end-diastolic volume index, *LVESVI* left ventricular end-systolic volume index, *LVEF* left ventricular ejection fraction, *LVMI* left ventricular mass index, *RWT* relative wall thickness, *TPM* trabeculae and papillary muscles

There were no significant interactions between CMR variables (including measures of myocardial remodeling such as LVMI and LVM/LVEDV ratio) and type of prevention (primary vs. secondary), QRS duration, or presence of LGE in our cohort (all interaction, p > 0.20). However, in patients with an ischemic heart disease a stronger effect was observed for lower LVEF when including TPM (*p* for interaction = 0.017), and for higher LVEDVI including and excluding TPM (*p* for interaction = 0.012 and 0.013, respectively), and higher LVESVI when excluding TPM (*p* for interaction = 0.0012), when compared with patients without ischemic heart disease.

## Discussion

In this 2-center study of 372 patients, we found a higher rate of appropriate ICD shocks and mortality in patients with lower LVEF, and that both LVEF and LVMI have independent prognostic value after adjusting for clinically relevant variables (such as age, type of prevention, ischemic etiology and presence of LGE), regardless of the post-processing method used. These findings suggest that either method can be used for risk stratifying ICD patients in clinical practice. When specifically evaluating LVEF cutoff values that are used commonly as criteria for ICD treatment, the hazard ratios for appropriate ICD shocks or death for LVEF below 30% and 35% were similar for both contouring methods. This implies that both methods provide similar prognostication in the ICD patient population.

Our study cohort included ICD recipients for primary and secondary prevention. Among the primary prevention cohort only, both segmentation methods for LVEF calculation had independent prognostic value for appropriate ICD shocks and all-cause death, similar to the entire cohort. There was no significant effect modification by the type of prevention (primary vs. secondary) on CMR predictor variables. These findings suggest that our results are applicable to a broad population of ICD recipients, regardless of ICD indication.

LVEF is currently the main parameter used for clinical SCD risk stratification. The role of LVEF as a prognostic factor in the ICD population has been well documented [17–19]. As expected, the post-processing method including the TPM in the total LVM resulted in higher LVEF values. However, no significant difference was found in the prognostic value between both CMR post-processing methods, which showed very similar strength of association, suggesting that the relationship between LVEF and our composite outcome remains similar regardless of the contouring method used. However, the overall prediction of ICD shocks or death was poor, with Somers' D values in the lower ranges (0.1–0.2) and C-statistics < 0.6. This is most likely due to the fact that LVEF may be an inaccurate predictor on its own, with some patients with low ejection fraction values never presenting with ICD shocks after ICD implantation. Whether each institution decides to include or exclude TPM from the total LVM, the method used should be consistent and clearly stated in the report, for the clinician's reference.

A stronger prognostic effect of higher LV volumes was observed in patients with ischemic compared with non-ischemic cardiomyopathy. Patients with a previous infarct are more likely to develop adverse myocardial remodeling at later stages of disease, especially those with extensive areas of myocardial scarring, with larger end-systolic and end-diastolic volumes. Our results suggest that LV enlargement secondary to infarct expansion and remodeling may confer a worse prognosis than the same degree of LV dilatation in non-ischemic cardiomyopathy. The explanation for this is likely multifactorial, including the fact that patients with ischemic heart disease may have a greater arrhythmogenic substrate than those with a non-ischemic etiology.

LVM was found to be an independent predictor for appropriate ICD shocks and all-cause mortality in our population. However, when assessed in the same model with LVEF, LVM did not have predictive value beyond LVEF. Previous studies have demonstrated LVM is a predictor of heart failure, coronary artery disease and stroke [20–22], and is independently associated with all-cause mortality and SCD [23]. Yet, most analyses of the predictive value of LVM measurements have been based on echocardiography. One of the strengths of our study is that all measurements were made on CMR, which has several advantages over echocardiography, including the lack of geometric assumption and acoustic window dependency, and better definition of the myocardial contours. In our study, we showed a clear association between LVEF and LVMI measured using CMR and appropriate ICD therapy and mortality. Our findings suggest a trend that LVMI may add some predictive value in patients with low LVEF, and this may deserve further study.

In evaluating LV geometry, because LV sphere volumes and LV sphericity were not independently associated with our primary outcome, it is unlikely they provide incremental prognostic value. In patients with advanced cardiomyopathies, myocardial remodeling is characterized by end-stage LV hypertrophy, a change in LV shape towards a more spherical configuration and reduced myocardial performance [24]. Furthermore, a relationship between LV shape and mortality has been described previously. Using echocardiography, LV sphericity measured by biplane method was found to be a predictor of 10-year survival in patients with acute ST elevation myocardial infarction [25]. More recently, a relationship with life-threatening ventricular arrhythmias has also been described [14, 26]. The use of CMR to evaluate the prognostic value of LV sphericity is fairly recent [27, 28], and only one study has analyzed the use of CMR-derived LV volumetric and geometric parameters to assess mortality in the ICD patient cohort, where LV sphericity index was reported to be a strong independent predictor of appropriate ICD therapy in patients with low LVEF [29]. In our study, we used the same method for measuring LV sphericity, but did not reach the same results. We did observe a significant association between higher LV sphericity values and increased mortality. Our two-center study had a larger patient cohort and robust CMR data, and our cohort may be more representative of the general population, with a broader spectrum of LV geometry.

Similarly, measurements of LV remodeling such as RWT and LV M/V ratio did not show significant prognostic value. In a select subgroup of patients with mild heart failure and left bundle branch block, RWT measured on echocardiography was significantly associated with ventricular arrhythmias and death in patients with cardiac resynchronization therapy-defibrillator [30]. Our study evaluated CMR-derived RWT measurements (likely a more accurate assessment of eccentric hypertrophy, owing to superior endocardial and epicardial definition when compared to echocardiography) and we studied a more heterogeneous cohort. Larger studies may be needed to clarify the prognostic value of RWT measurements by CMR in a broader ICD population.

The clinical implications of this study are multiple. The absence of a significant difference in prognostic value between the two proposed methodologies for LVEF measurement by CMR is relevant because of the current variability and lack of consensus between centers on which is the best post-processing technique. Our results showed very good inter-observer agreement (> 0.95) and support the notion that each institution may use the method of their preference, likely the one with less inter-observer variability. Our data also suggest LVM (which can also be assessed on non-contrast CMR) may improve risk stratification in this population and better target the use of ICD therapy.

## Limitations

This study has several limitations. Our population included only patients who received an ICD as part of their clinical treatment; therefore, our results may not be applicable to patients with higher LVEF, who would not get a subsequent ICD implant. To enhance the generalizability of our findings, we included all evaluable CMR studies from consecutive ICD recipients, except those patients whose LVEF was not the main determinant for ICD implant decisions (e.g. channelopathies). However, there might still be a selection or referral bias in our study population. The majority of our study population were men (79%), which could represent some selection bias, and the evaluation of sex-based differences [31] is beyond the scope of this study. Finally, the low predictive ability of our multivariable models may reflect the challenges of predicting arrhythmic events in the ICD population, and even though model performance was slightly increased when including LVEF and LVMI in the same models, which may suggest a modest incremental prognostic value attributable to LVMI, our study may not be adequately powered to rigorously assess this.

## Conclusion

Our study demonstrated similar prognostic value between the two CMR post-processing methods for calculating LVEF, whether TPM were included (trabeculated endocardial contours) or excluded (rounded endocardial contours) from the total LVM. Accordingly, the best approach may simply be the most reproducible one in each institution, and the method used should be specified in the final report. Measures of LV sphericity did not add incremental prognostic value. Finally, LVMI and LVEF showed independent prognostic value for appropriate ICD shocks and mortality in the ICD population. These findings suggest that assessment of LVM by CMR may be a useful additional tool for clinical decision-making in candidates for ICD therapy, specifically in those with low LVEF.

## Data Availability

The datasets generated and/or analysed during the current study are not publicly available due to requirement for data sharing agreement but are available from the corresponding author on reasonable request.

## Abbreviations

BSA: Body surface area
bSSFP: Balanced steady-state free precession
CI: Confidence interval
CMR: Cardiovascular magnetic resonance
ECG: Electrocardiogram
FA: Flip angle
FOV: Field of view
HR: Hazard ratio
ICD: Implantable cardioverter defibrillator
IQR: Interquartile range
LGE: Late gadolinium enhancement
LV: Left ventricle/left ventricular
LVEDV: Left ventricular end-diastolic volume
LVEDVI: Left ventricular end-diastolic volume index
LVEF: Left ventricular ejection fraction
LVESV: Left ventricular end-systolic volume
LVESVI: Left ventricular end-systolic volume index
LVM: Left ventricular mass
LVMI: Left ventricular mass index
M/V: Mass/volume ratio
RWT: Relative wall thickness
SCD: Sudden cardiac death
SD: Standard deviation
SV: Stroke volume
TE: Time to echo
TPM: Trabeculae and papillary muscles
TR: Repetition time
